# The Relationship between Regional Gray Matter Volume of Social Exclusion Regions and Personal Self-Esteem Is Moderated by Collective Self-Esteem

**DOI:** 10.3389/fpsyg.2017.01989

**Published:** 2017-11-17

**Authors:** Xin Wu, Yujie Chen, Bing Chen, Lili Guan, Yufang Zhao

**Affiliations:** ^1^Key Laboratory of Cognition and Personality (SWU), Ministry of Education, Chongqing, China; ^2^Faculty of Psychology, Southwest University, Chongqing, China; ^3^School of Psychology, Northeast Normal University, Changchun, China

**Keywords:** personal self-esteem, collective self-esteem, social exclusion, voxel-based morphometry, regional gray matter volume

## Abstract

According to sociometer theory, self-esteem is an internal monitor of positive social bonds to others. Social exclusion can break or threaten social bonds, which might be reflected by the brain structure of social exclusion regions. Thus, self-esteem might be influenced by structurally individual differences in these regions. It has been suggested that self-esteem can be divided into personal (PSE) and collective (CSE) self-esteem and CSE can bring individuals many benefits, such as acceptance, belonging, and social support, which could further maintain or increase their PSE. Based on this, we hypothesized that CSE might moderate the relationship between structurally individual differences in social exclusion regions and PSE. Therefore, in the present study, the moderating effect of CSE on the relationships between PSE and individual differences in regional gray matter volume (rGMV) of 10 social exclusion regions from previous meta-analysis of social exclusion were investigated using voxel-based morphometry. The results showed that CSE played a moderating role in the relationship between PSE and rGMV of the left posterior cingulate cortex (PCC). Specifically, PSE was positively associated with rGMV of left PCC in lower CSE, while there was no significant relationship between PSE and rGMV of left PCC in higher CSE. Therefore, we believe that compared with a higher CSE, because of lack of acceptance, belonging, and social support from valued groups, lower CSE individuals might be more prone to be influenced by social exclusion with decreased rGMV of the left PCC, which makes them more prone to develop lower PSE.

## Introduction

Self-esteem refers to an attitude formed by self-evaluation based on positive and negative aspects of oneself (Rosenberg, 1965; Brown, 1993; Baumeister et al., 1998). Based on sociometer theory, self-esteem is an internal monitor of the degree to which individual is accepted and included by others vs. rejection and exclusion (Leary and Baumeister, 2000; Leary et al., 2001). In other words, self-esteem is an internal monitor of the information regarding the quality of an individual’s social bonds to others (Leary, 1999). Social exclusion refers to the phenomenon that an individual’s existing or potential social bonds are broken or threatened (Kawamoto et al., 2015). Moreover, previous studies found that individuals who chronically experience real or imagined rejection are prone to develop lower self-esteem relative to individuals that feel accepted and included in their social environment (Dandeneau and Baldwin, 2004; Zadro et al., 2004).

Previous studies uncovered the association between social exclusion and many brain regions. Eisenberger et al. (2003) found that the dorsolateral anterior cingulate gyrus (dACC) is more active when individuals are excluded rather than included in a Cyberball game and the self-reported social distress was positively predicted by activity level of the dACC. In succeeding studies, the critical role of dACC was repeatedly confirmed (Eisenberger et al., 2007, 2011; Onoda et al., 2009, 2010; DeWall et al., 2012). It seems that the dACC is a core brain region of social exclusion. However, it has been suggested that social exclusion in Cyberball game is mixed with violations of expectation for inclusion and the dACC might be related to cognitive conflicts of violations of expectation (Somerville et al., 2006). Consistent with this perspective, previous studies found that both the dACC and the ventral anterior cingulate gyrus (vACC) were more active under social rejection, but only the vACC was not influenced by violations of expectation (Somerville et al., 2006; Gyurak et al., 2011). Moreover, previous meta-analyses of studies of social exclusion found that there is no robust evidence to support the notion that dACC is the core region of social exclusion (Cacioppo et al., 2013; Vijayakumar et al., 2017). Whereas, to discriminate brain regions for social exclusion and violations of expectation using the Cyberball game, Kawamoto et al. (2012) found that activation of the dACC might be specifically involved in social exclusion (Rotge et al., 2015). Furthermore, Rotge et al. (2015) divided the anterior cingulate gyrus (ACC) into the anterior (aMCC) and posterior (pMCC) midcingulate cortex, as well as the pregenual (pgACC) and subgenual (sgACC) subdivisions, and found that the aMCC, pgACC, and sgACC are all involved in social exclusion through a meta-analysis of the contribution of the ACC to social pain. In addition to the ACC, the ventral prefrontal cortex (VLPFC), the anterior insular (AI), and the posterior cingulate cortex (PCC) have been also found to be involved in social exclusion (e.g., Eisenberger et al., 2003; Somerville et al., 2006).

According to the studies mentioned above, the social exclusion regions in the present study refer to the ACC (aMCC, pgACC, sgACC), AI, and VLPFC. In addition, it has found that self-esteem is associated with activation of some of these regions induced by social exclusion. For example, Eisenberger et al. (2011) found that AI and aMCC were negatively correlated with self-esteem; and aMCC, pgACC, and AI were more active for individuals with lower self-esteem relative to individuals with higher self-esteem when experiencing social exclusion (Onoda et al., 2010). According to Leary and Baumeister (2000), self-esteem results from past experiences of being rejected or included, and individuals with lower quality relationships with family members, friends and romantic partners, also show lower levels of self-esteem (Denissen et al., 2008). Although no direct evidence for social exclusion has found employing the same tasks used in the above functional MRI studies, numerous studies have shown the neurobiological effects of these social bonds to others on structures of brain regions, including social exclusion brain regions, such as the positive effects of positive parenting on the brain structure of the ACC (Rao et al., 2010; Whittle et al., 2014) and the deleterious effects of adverse friendships on brain structure of the ACC (Tomoda et al., 2009). Based on these observations, the structure of these social exclusion regions could be shaped by social exclusion to some extent, which might imply that individual differences in the brain structure of these regions might be related to the degree of influence of social exclusion. Therefore, we speculated that self-esteem might be associated with individual differences in the brain structure of some parts of the social brain regions.

According to social identity theory (Tajfel and Turner, 1979; Turner et al., 1979; Tajfel, 1982; Turner, 1982), an individual’s self-concept has two different aspects: personal identity and social identity. Correspondingly, self-esteem can be divided into personal self-esteem (PSE) and collective self-esteem (CSE). The self-esteem mentioned above is PSE, which reflects the tendency of positive self-evaluation based on a personal level, while CSE refers to the generalized tendency to evaluate one’s social identity positively (Luhtanen and Crocker, 1992). It has been found that PSE and CSE are positively correlated, because they both have a shared core in self-concept and ensure the positivity of the self-concept as a whole (Luhtanen and Crocker, 1992). According to Crocker and Luhtanen (1990), CSE can also be seen as positive identification with valued social groups. Affiliation to valued social groups can bring individuals many benefits, such as acceptance, belonging, and social support (Stephan and Mealy, 2011), and it can also increase their PSE (Crocker and Luhtanen, 1990). From this perspective, despite social exclusion regions having effects on PSE, relative to individuals with lower CSE, individuals with higher CSE could acquire more acceptance, belonging, and social support from the valued social groups they belong to, which might reduce the effects of these regions on their PSE. Thus, we speculated that CSE might play a moderating role in the relationship between PSE and individual differences in the structure of social exclusion regions.

The aim of the current study was to investigate whether the relationship between PSE and the structure of social exclusion regions was moderated by CSE, using voxel-based morphometry (VBM). VBM is widely used to measure the brain structure in structural magnetic resonance imaging. Moreover, the regional gray matter volume (rGMV) in certain brain region ensures that specific cognitive tasks are performed more efficiently (Haier et al., 2004; Colom et al., 2009) and ability training can shape the rGMV in certain regions related to this ability (Gaser and Schlaug, 2003; May and Gaser, 2006). Therefore, in the present study, the moderating effect of CSE on the relationship between individual differences in the rGMV of social exclusion regions and PSE was investigated to verify our hypothesis.

## Materials and Methods

### Ethics Statement

This study was approved by the ethical standards of the Brain Imaging Center Institutional Review Board of Southwest China University and complied with the standards of the Declaration of Helsinki (1991). All subjects gave their written informed consent. This study was carried out in accordance with the recommendations of ‘Southwest University MRI lab of guidelines, Faculty of Psychology of committee.’ We have followed the guidance of the APA requirements of human subjects.

### Subjects

One hundred and forty-five undergraduate students (67 men and 78 women) aged 19–26 years (mean age = 22.7 years) participated in the experiment as paid volunteers. All were right-handed, had no current or past neurological or psychiatric illnesses, and had normal or corrected-to-normal vision.

### Materials

The collective self-esteem scale (CSES) (Luhtanen and Crocker, 1992) was used to measure the CSE. The Chinese version of the CSES was translated from the CSES and revised by Jia (2009). Similar to the English CSES (Luhtanen and Crocker, 1992), the Chinese version of the CSES has 16 items that are divided into four factors (membership, private, public, and identity). Subjects are asked to rate each item on a 7-point scale (from 1 = totally disagree to 7 = totally agree). In the Chinese version of the CSES, the Cronbach’s α of the subscales and the total scale range were from 0.69 to 0.84. In our sample, the CSES had satisfactory internal consistency: Cronbach’s α = 0.819, and its mean score was 79.69, with a standard deviation of 10.25.

The Rosenberg self-esteem scale (RSES) was used to measure the PSE. The RSES consists of 10 items, and is widely used as a measure of global trait self-esteem (Pan et al., 2015; Yang et al., 2016; Peng et al., 2017). Participants are asked to rate each item on a 4-point scale (from 1 = totally disagree to 4 = totally agree). The scores of the five negatively worded items are reversed and the total self-esteem score is found by summing the 10 responses. In our sample, the 10-item RSES had satisfactory internal consistency (Cronbach’s α = 0.896), and its mean score was 28.71, with a standard deviation of 5.05.

### Structural Magnetic Resonance Imaging (MRI) Data Collection and Preprocessing

Imaging was performed on a 3-T Siemens Tim Trio scanner equipped with a 32-channel head coil. A whole-head high-resolution structural scan of each participant was performed using a T1-weighted MPRAGE sequence (repetition time = 1900 ms, echo time = 2.52 ms, inversion time = 900 ms, flip angle = 9 deg, resolution matrix = 256 × 256, slices = 176, thickness = 1.0 mm, voxel size = 1 mm × 1 mm × 1 mm).

The MRI data were processed using SPM 8 (Statistical Parametric Mapping, Wellcome Trust Centre for Neuroimaging, United Kingdom) and the VBM 8^1^ Toolbox implemented in MATLAB R2013a, according to the VBM 8 Toolbox Manual. The preprocessing steps included bias-field correction and segmentation into gray matter (GM), white matter (WM), and cerebrospinal fluid (CSF). Specifically, the individual T1 images were overlaid on modified versions of the International Consortium for Brain Mapping (ICBM) tissue using a non-linear deformation field estimation, and segmented into GM, WM, and CSF. The T1 images were then normalized to a template space (Montreal Neurological Institute, MNI) using high dimensional DARTEL normalization. Before entering the GM images into a statistical model, the image data were smoothed using a Gaussian kernel of 8-mm^3^ full-width at half maximum.

### Statistical Analysis

#### Region of Interest (ROI) Analysis

To investigate the moderating effect of CSE on the relationship between PSE and rGMV of social exclusion brain regions, 10 ROIs were created based on meta-analyses studies of social exclusion (Cacioppo et al., 2013; Rotge et al., 2015; Vijayakumar et al., 2017). Specifically, based on the results of these studies, the left inferior frontal gyrus (IFG), bilateral pgACC, bilateral aMCC, bilateral AI, left sgACC, and the bilateral PCC were created around the center of the coordinates reported by these studies with an 8-mm radius using WFU PickAtlas Tool^2^ (see **Table 1**). The mean rGMV of these 10 ROIs was then extracted to analyze the relationship between PSES and the 10 ROIs, as well as the moderating effect of the total score of CSES on the relationship between rGMV of social exclusion brain regions and the total score of RSES using model 1 of PROCESS 2016 for SPSS (Hayes, 2016). The significance criterion for both the correlation and moderating analysis were *p* < 0.05 with Bonferroni correction, which means that 0.05 was divided by number of ROIs and the significant criterion in this study was *p* < 0.005.

**Table 1:** Region of interest (ROIs) of social exclusion.

	Left	Right
aMCC	[–8, 18, 42]	[8, 24, 24]
pgACC	[–4, 42, 20]	[4, 36, -4]
sgACC	[–8, 18, -6]	
PCC	[–8, -56, 12]	[6, -40, 40]
IFG	[–46, 32, -10]	
AI	[–36, 20, -10]	[38, 18, -6]

#### Whole Brain Analysis

Although we hypothesized that the social rejection brain regions have an important role in the individual differences in PSE, there might be other mechanisms of PSE. Thus, whole brain analysis was used to find the brain regions, except for the social rejection brain regions. In the whole brain analysis, the gray images were entered into a multiple linear regression analysis implemented in SPM8 to determine the relationship between the rGMV and the total score of CSES and RSES. We used an absolute threshold mask by including voxels with a gray or WM value greater than 0.2. To remove potential confounders, the total intracranial volume, age, and sex were controlled in the analyses as nuisance covariates. For the all analyses, the significance criterion was the false discovery rate (FDR)-corrected *p* < 0.05 with 50 voxels.

## Results

### Results of Moderator Analysis

**FIGURE 1:**
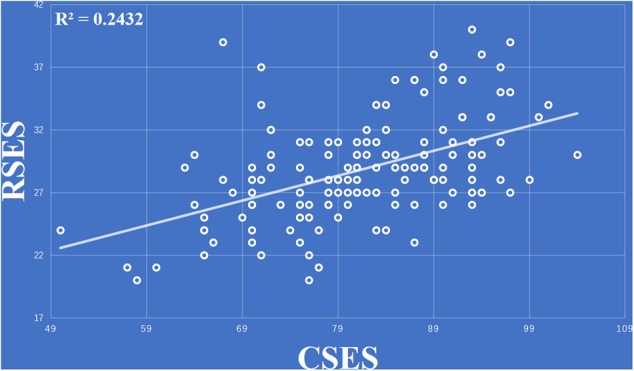
Total scores of the collective self-esteem scale (CSES) were positively correlated with total score of the Rosenberg self-esteem scale (RSES).

The data for one the subjects was discard because the score of the RSES was lower than the mean score of RSES minus three standard deviations. The behavior results showed that total scores of the CSES were positively correlated with the total score of the RSES (*r* = 0.493, *p* < 0.001) (**Figure 1**).

After controlling for whole brain volume, age, and gender, the negative relationship between the RSES and the right AI (*r* = -0.239, *p* = 0.004) was significant, while the negative relationship between the RSES and the left pgACC (*r* = -0.219, *p* = 0.008) was marginally significant (**Table 2**).

**Table 2:** The relationship between rGMV of ROIs and CSES, PSES.

		lPCC	rPCC	lIFG8	rpgACC	raMCC	rAI	lsgACC	lpgACC	laMCC	lAI
CSES	*r*	–0.137	–0.156	–0.067	–0.132	–0.090	–0.187	–0.070	–0.130	–0.090	–0.094
	*p*	0.104	0.063	0.426	0.117	0.283	0.025	0.403	0.122	0.287	0.263
PSES	*r*	–0.029	–0.110	–0.053	–0.146	–0.152	***-0.239***	–0.183	**-0.219**	–0.166	–0.065
	*p*	0.730	0.191	0.532	0.081	0.070	***0.004***	0.028	**0.008**	0.048	0.443

*The bolded and italic values meant that the relationship was significant at *p* < 0.05 with Bonferroni correction and the bolded values meant that the relationship was marginally significant at *p* < 0.05 with Bonferroni correction.*

**FIGURE 2:**
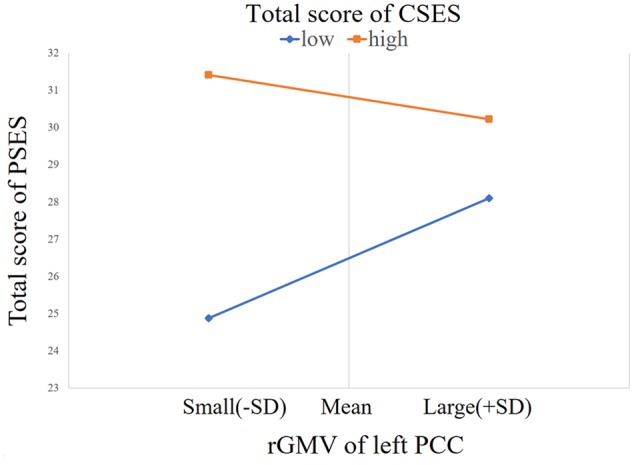
The relationship between PSE and the rGMV of the left PCC was moderated by CSE [contrast values were derived from the left PCC area and predicted values were graphed at 1 standard deviation (SD) above and below the respective means of the centered predictors].

Process Model 1 moderator analysis showed that the interaction between the left PCC and total CSES scores was significant [*β* = -0.25, *F*(1,139) = 10.33, *p* = 0.002, Δ*R*^2^ = 0.051]. There were no interactions between other regions and the total CSES scores [vACC: *β* = -0.165, *F*(1,139) = 0.22, *p* = 0.639, Δ*R*^2^ = 0.001; right PCC: *β* = -0.165, *F*(1,139) = 0.22, *p* = 0.639, Δ*R*^2^ = 0.001; right pgACC: *F*(1,139) = 0.465, *p* = 0.496, Δ*R*^2^ = 0.002; right pgACC: *F*(1,139) = 2.037, *p* = 0.156, Δ*R*^2^ = 0.010; right aMCC: *F*(1,139) = 0.014, *p* = 0.905, Δ*R*^2^ = 0.001; left aMCC: *F*(1,139) = 3.465, *p* = 0.065, Δ*R*^2^ = 0.018; right AI: *F*(1,139) = 0012, *p* = 0.913, Δ*R*^2^ = 0.0001; left AI: *F*(1,139) = 3.234, *p* = 0.074, Δ*R*^2^ = 0.017; left sgACC: *F*(1,139) = 0.052, *p* = 0.821, Δ*R*^2^ = 0.003]. Simple slope test analysis showed that for the lower total CSES score (mean - 1SD), the rGMV of the left PCC was positively correlated with the total RSES score [β = 0.35, *t* = 2.90, *p* = 0.004], while for the higher total CSES score (mean + 1SD), the relationship between rGMV and total RSES score was not significant [β = -0.15, *t* = -1.56, *p* = 0.122] (**Figure 2**).

### Results of Whole Brain Analysis

**FIGURE 3:**
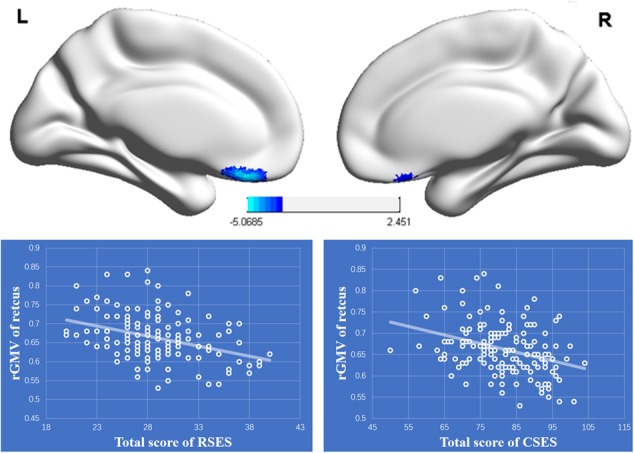
**Upper:** The regional gray matter (rGMV) was negatively associated with personal self-esteem (PSE). The significant cluster is shown that exceeded the threshold *p* < 0.05 FDR-corrected with 50 voxels. **Lower**: The negative relationships between the rGMV of the left rectus and total scores of RSES and CSES.

The results of whole brain analysis showed that the rGMV of the rectus (-8, 35, -21) was negatively correlated with total score for RSES (see **Figure 3**), while there were no regions whose rGMV was significantly associated with total score of the CSES. However, ROI analysis showed that the rGMV of the rectus was also negatively correlated with total score of the CSES (*r* = -0.322, *p* < 0.001) (see **Figure 3**). The moderating role of CSE in the relationship between PSE and the left rectus was also analyzed, and the results showed that the interaction between CSE and the left rectus was not significant [*F*(1,139) = 0.004, *p* = 0.951, Δ*R*^2^ < 0.001].

## Discussion

In the present study, the moderating role of the rGMV of social rejection brain regions in the relationship between CSE and PSE was investigated using VBM. Based on a previous meta-analysis of fMRI studies in social exclusion (Cacioppo et al., 2013; Rotge et al., 2015; Vijayakumar et al., 2017), we analyzed the moderating role of CSE in the relationship between PSE and rGMV of 10 ROIs observed by social exclusion studies. The ROI results showed that the relationship between the rGMV of the left PCC and PSE was moderated by CSE. Specifically, the rGMV in the left PCC was positively associated with PSE in lower CSE individuals, while there was no significant correlation between PSE and the rGMV of the left PCC in higher CSE individuals. However, our results also showed that the negative relationships between the right AI and the left sgACC and PSE were not moderated by CSE. In addition, according to our results of whole brain analysis, both the CSE and PSE were negative correlated with the left rectus in the vmPFC, and the relationship between PSE and left rectus was also not influenced by CSE.

It has been observed that the brain structure of the PCC is influenced by social bonds to others, such as higher-quality parental care early in childhood increasing the rGMV of the PCC (Rao et al., 2010). Social exclusion can break or threaten an individual’s existing or potential social bonds (Kawamoto et al., 2015) and the activation of the left PCC, which is a brain region related to social exclusion, as it is repeatedly detected by experimental studies (Onoda et al., 2009; Bolling et al., 2011) and a meta-analysis of social exclusion studies (Vijayakumar et al., 2017). Thus, our results confirmed that the relationship between social exclusion region in the PCC and PSE was moderated by CSE.

Although self-concept is stable in some respects, it also has malleability to some extent under most circumstances, which means that self-concept could be revised or reorganized to take on new characteristics (Markus and Kunda, 1986; Slotter and Gardner, 2012). Richman et al. (2015) confirmed that social exclusion could increase self-concept malleability and make self-concept more similar to the excluders. The left PCC might be related to increased malleability of self-concept under social exclusion circumstances because of its role in self-referential processing. Previous studies have repeatedly found that PCC is involved in self-referential processing or self-reflection (Johnson et al., 2006; Schneider et al., 2008; van der Meer et al., 2010). Northoff and Bermpohl (2004) suggest that the PCC is involved in “integrating self-referential stimuli in the context of one’s own person.” Thus, we thought that PCC might be involved in self-knowledge gathering in processes of forming similar self-concept of excluders when they were excluded. People have a fundamental need for social belonging and connection, and increased self-concept malleability could provide individuals with opportunities to regain social connection or gain renewed affiliation (Richman et al., 2015). Based on these, we thought that the moderating roles of CSE in the relationship between the left PCC and PSE might reflect the fact that high levels of GM in this region could buffer the loss of CSE and maintain the PSE. Specifically, although individuals with lower CSE acquire less acceptance, belonging, and social support from their ingroups, relative to individuals with higher CSE, with an increasing rGMV of the PCC, individuals are more prone to increase their self-concept malleability and could develop a higher PSE, similar to individuals with a higher CSE.

In addition, personality systems interactions theory (PIST) suggests that positive interaction experiences during early childhood lead to the formation of a neuropsychological structure, the integrated self (e.g., Kuhl, 2000; Schore, 2002; Kuhl et al., 2015). It has been found that the PCC has dense structural connections to many other brain regions (Hagmann et al., 2008). Previous studies of functional connectivity also found that the PCC acts as a cortical hub, which means that the PCC is a highly connected region for cortical and subcortical networks, and is involved in broad information gathering (Hagmann et al., 2008; Leech et al., 2012). Considering the role of the left PCC in self-referential processing (Johnson et al., 2006; Schneider et al., 2008; van der Meer et al., 2010), it might be an important part of the neural underpinning of the integrated self. Furthermore, predictive and reactive control systems theory (PARCS) suggests that the PCC is part of the “predictive system” that constructs internal models of social support resources and positive self-esteem, and utilizes those models in prospective cognition and behavior (Tops et al., 2014a,b). According to PIST and PARCS, our results might reflect the fact that PSE loss for individuals with lower CSE could be buffered by the integrated self, even in the absence of social support resources, which also means that if the integrated self is less developed (smaller rGMV of left PCC), individuals with more resources of acceptance, belonging, and social support (higher CSE) could also reduce their PSE loss.

It has been suggested that PSE could buffer negative emotions (Holland et al., 2002; Brown, 2010), such as sadness, anger, shame, as well as other intensely negative emotions (Crocker, 2002; Crocker and Park, 2004). The role of PSE in negative emotions could be supported by our results, which found that the rGMV of both the right AI and the left pgACC decreased with increasing PSE. As Rotge et al. (2015) pointed out in their review, many previous studies found that the pgACC is involved in social pain induced by social exclusion, and the results of their meta-analysis also showed that the pgACC is related to social pain. Moreover, numerous studies also found that the right AI is related to self-reported distress during social exclusion (Eisenberger et al., 2003, 2007; Cacioppo et al., 2013). Thus, the negative relationships between PSE and these regions might reflect the fact that individuals with lower PSE could be more prone to being influenced by the negative emotions (such as social pain) induced by social exclusion. In consideration of the relationships being not moderated by CSE, the effects of negative emotions on PSE might be more difficult to buffer by the benefits (such as social support resources) of CSE.

According to social identity theory (Tajfel and Turner, 1979; Turner, 1982), people’s definition of themselves result from their memberships of various social groups or categories to some extent. Moreover, as social identity contributes to self-conception (Luhtanen and Crocker, 1992), it also contributes to self-evaluation or self-esteem (Pelham and Swann, 1989; Luhtanen and Crocker, 1991, 1992). It has been found that PSE and CSE are positively correlated, because they both have a shared core in self-concept and ensure the positivity of the self-concept (Tajfel and Turner, 1986; Luhtanen and Crocker, 1992). From this perspective, the positive relationship between PSE and CSE might imply that the shared core in self-concept might result from social groups or categories. Thus, the neural basis of the relationship between PSE and CSE might reflect the collective source of self-concept, which might be related to the left rectus in the vmPFC. Previous studies had repeatedly found that the vmPFC was involved in self-referential processing and self-judgments (Northoff et al., 2006; Sui et al., 2013). Moreover, it was confirmed that the rGMV of the orbital part in the vmPFC was reduced for interdependent self-construal (Kitayama et al., 2017; Wang et al., 2017). Considering that individuals with interdependent self-construal tend to view themselves as a part of groups (Sato and Cameron, 1999), we thought that the decreased rGMV of the left rectus with increases in both PSE and CSE might reflect that the shared core of PSE and CSE results from interdependent self-construal.

There is inconsistent evidence regarding the neural mechanism of PSE. In the present study, the results showed that PSE is negatively associated with the left rectus. A previous study found that PSE was positively related with certain other brain regions, such as the ACC, the left lateral prefrontal cortex, the right hippocampus, and the left hypothalamus (Agroskin et al., 2014). Although there are some differences between our study and that of Agroskin et al. (2014) such as the sample size and the correction method for the results, which could give rise to difference in statistical power and poor reproducibility (Button et al., 2013; Dubois and Adolphs, 2016), the inconsistent results obtained in the present study and those reported by Agroskin et al. (2014) may not be solely caused by the sample size and correction method, but may involve more complex factors. First, we thought that the negative relationship between PSE and the left rectus observed in this study might be related to interdependent self-construal, which could appear in non-Western collectivistic countries, such as Japan (Sato and Cameron, 1999). Thus, we thought that the inconsistent results might reflect different culture contexts between the subjects of these two studies, such as the subjects of Agroskin et al.’s study coming from the University of Salzburg (Western individualistic country) and the subjects of our study coming from Southwest China University (non-Western collectivistic country). In addition, the mean of the PSE in the present study was 2.8 (28.7/10), whereas that for the previous study was 3.4, and the difference of the mean scores might be also responsible for the inconsistent results. Thus, the relationship between PSE and the structure of these brain regions should be verified further using a larger sample size and datasets from different countries, with correction methods, in a future study.

## Author Contributions

XW and YC are contributed equally. XW, YC, and YZ designed this research and wrote this paper. XW and BC analysis the data. XW, LG, and YC collected the data.

## Conflict of Interest Statement

The authors declare that the research was conducted in the absence of any commercial or financial relationships that could be construed as a potential conflict of interest.

## Footnotes

1 http://dbm.neuro.uni-jena.de/vbm8/

2 http://www.nitrc.org/projects/wfu_pickatlas/
